# Exploring the Interplay of Lattice Dynamics and Charge Transport in Organic Semiconductors: Progress Toward Rational Phonon Engineering

**DOI:** 10.1002/anie.202507566

**Published:** 2025-05-29

**Authors:** Barbara M. T. C. Peluzo, Rahul Meena, Luca Catalano, Guillaume Schweicher, Michael T. Ruggiero

**Affiliations:** ^1^ Department of Chemistry University of Rochester Rochester NY 14627 USA; ^2^ Université Libre de Bruxelles (ULB) Faculté des Sciences Laboratoire de chimie des polyméres Boulevard du Triomphe Bruxelles 1050 Belgium; ^3^ Department of Life Sciences University of Modena and Reggio Emilia Modena 41125 Italy; ^4^ Department of Chemical Engineering University of Rochester, Rochester NY 14627 USA

**Keywords:** Materials, Organic electronics, Phonon, Semiconductor, Terahertz

## Abstract

Organic semiconductors (OSCs) have garnered significant attention due to their potential use in flexible, lightweight, and cost‐effective electronic devices. Despite their promise, the assembly of organic molecules into the condensed phase promotes a diverse set of lattice dynamics that introduce a detrimental modulation in the intermolecular electronic structure—termed dynamic disorder—that results in charge carrier mobilities that are orders of magnitude lower than inorganic semiconductors. This dynamic disorder is generally associated with low‐frequency phonons, yet whether a small subset of modes or a broad range of phonons  drives dynamic disorder remains contested. Resolving this debate is critical for defining how targeted phonon engineering could practically improve OSC performance. In this review, we explore progress toward uncovering the interplay between lattice dynamics and charge transport in OSCs, focusing on the critical role of thermally activated phonons. We describe the powerful insight that mode‐resolved analyses of electron–phonon interactions lends toward the rational design of new materials. We highlight recent efforts to achieve this, showcasing proposed strategies to mitigate dynamic disorder through molecular and crystal design. This work offers an overview of the insight gained toward understanding the fundamental mechanisms governing charge transport in OSCs and outlines pathways for enhancing performance via targeted manipulation of interatomic/intermolecular interactions and resulting phonon modes.

## Graphical abstract

This Minireview highlights recent advances in understanding the role of lattice dynamics and electron–phonon coupling in organic semiconductors. Emphasis is placed on mode‐resolved insights into dynamics‐induced electronic disorder, emerging strategies for phonon engineering, and the implications of both low‐ and high‐frequency vibrations on charge transport.

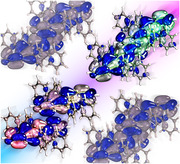

## 1 Introduction

Organic semiconductors (OSCs) represent a groundbreaking class of electronic materials that have transformative potential for applications ranging from flexible electronics to optoelectronic devices, including organic field‐effect transitors (OFETs), organic light‐emitting diodes (OLEDs), and organic photovoltaics (OPVs).^[^ ^1^, ^2^, ^3^, ^4^ ^]^ Unlike their inorganic counterparts, OSCs consist of carbon‐based molecules or polymers that exhibit semiconducting properties, in many cases, due to molecules that contain highly conjugated aromatic systems with delocalized π‐electrons.^[^ ^5^, ^6^ ^]^ The intrinsic properties of organic solids, such as mechanical flexibility, lightweight nature, and the possibility of low‐cost, large‐area manufacturing via solution processing, make OSCs highly attractive for next‐generation electronic devices that can leverage these in unique ways that more traditional inorganic materials cannot easily replicate.^[^ ^7^, ^8^, ^9^ ^]^ Furthermore, the development of new OSCs can utilize the extensive molecular and crystalline engineering tools that have been developed to readily modify molecular and/or bulk structures, which provide precise control and allow for promoting favorable features, opening up a nearly infinite chemical space for design and optimization.^[^ ^10^, ^11^, ^12^, ^13^ ^]^

However, the same factors that provide OSCs with their significant potential are also what reduce their applicability, primarily due to much lower charge carrier mobilities compared to inorganic semiconductors.^[^ ^14^, ^15^ ^]^ Charge carrier mobility (µ) is a measure of how fast charge carriers, such as electrons or holes, can move through a material per unit of applied electric field. High mobility is crucial for efficient charge transport, impacting the performance and efficiency of electronic devices. OSCs often exhibit much lower charge carrier mobilities, usually 1 – 20 cm^2^ ·V^−1^ ·s^−1^, compared to inorganic semiconductors (>10^3^ cm^2^ ·V^−1^ ·s^−1^). This originates from a variety of factors, most of which arise from their inherent molecular nature.

**Figure 1:**
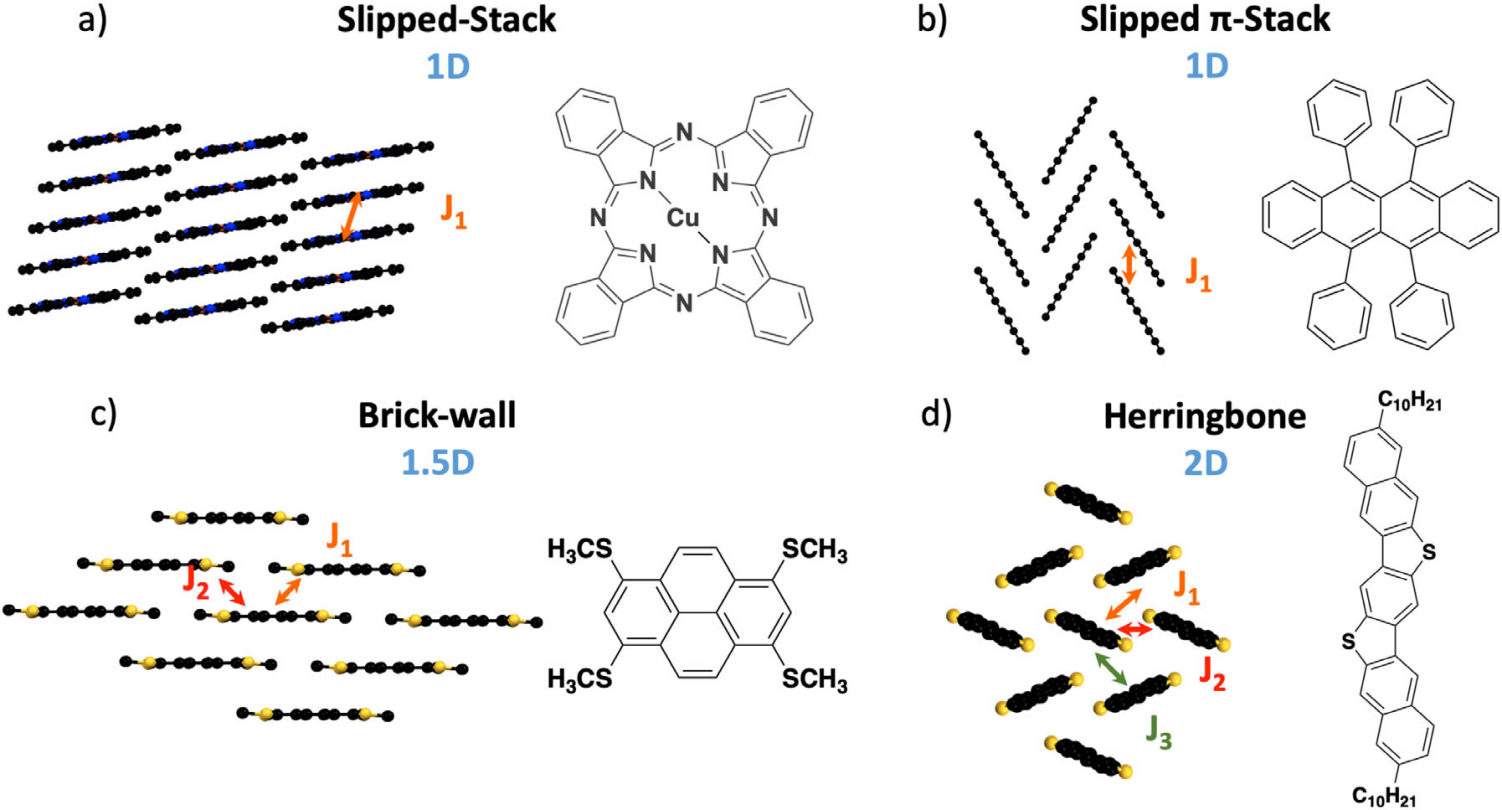
Examples of common packing motifs exhibited by OSCs and the dimensionality of the charge transport in each packing. (a) and (b) slipped‐stack and slipped π‐stack, respectively, where the charge transport is predominately uni‐dimensional due to a single major transfer integral *J*_1_ between pairs of molecules. c) Brick‐wall, where two major transfer integrals *J*_1_ and *J*_2_ arise due to the alternate arrangement of molecules in a single layer. d) Herringbone, which exhibits three major transfer integrals, *J*_1_, *J*_2_, and *J*_3_.

Inorganic semiconductors (e.g., crystalline silicon) are held together by robust covalent bonds, which promote efficient charge transport via two related, but distinct, mechanisms.^[^ ^16^, ^17^ ^]^ In the static regime, such solids have a large overlap of interatomic wavefunctions, providing an efficient route for charge transport.^[^ ^18^, ^19^ ^]^ Furthermore, the nature of the strong interatomic interactions promotes the formation of a stiff lattice, where nuclear displacements are minimized due to the associated high frequencies of the lattice vibrations.^[^ ^20^, ^21^, ^22^ ^]^ On the other hand, the bulk structures of OSCs are often characterized by molecules or polymers that are held together by non‐covalent interactions—examples of common OSC structural motifs are shown in Figure 1. This inherently leads to intermolecular wavefunction overlap that is reduced compared to covalently bound networks, and various routes for charge transport—indicated in Figure 1 by the symmetry‐independent transfer integrals, *J*.^[^ ^23^, ^24^, ^25^ ^]^ These weak interactions promote a large number of low‐frequency (terahertz or far‐IR) molecular dynamics that result in large‐amplitude nuclear motions, altering the population of these vibrational states at room temperature.^[^ ^26^, ^27^ ^]^ Such dynamics are incredibly detrimental to charge transfer, as they readily modulate the intermolecular charge transfer integral (*J*), which significantly hinders electronic coherence—and by extension, the charge carrier mobility— in OSCs.^[^ ^28^, ^29^, ^30^ ^]^

Over the past two decades, an increasing number of studies have shown that these thermally activated dynamics play a critical role in dictating charge transport in OSCs, and as such they represent one of the most active areas of study in the design of advanced organic materials for transistor applications.^[^ ^31^, ^32^, ^33^, ^34^, ^35^, ^36^ ^]^ Early efforts from Troisi and coworkers effectively studied the modulation of electronic structure in OSCs using molecular dynamics simulations^[^ ^30^, ^37^, ^38^ ^]^ In one seminal work, the intermolecular electronic coupling in pentacene‐derivative crystals was shown to be significantly modulated by thermally active dynamics, which resulted in localizing the free charge carriers to individual molecular sites and thereby reduced charge transport.^[^ ^37^ ^]^ In that study, the authors coined the now‐often used phrase of “dynamic disorder” to represent the fluctuations in the intermolecular coupling as a result of thermal motion. Over the years, this rationalization has proven to be effective, and has spurred numerous further studies related to characterizing thermally activated dynamics, including the incorporation of dynamic disorder into theoretical models to efficiently compute free‐carrier mobilities,^[^ ^28^, ^39^, ^40^, ^41^ ^]^ and, more recently, leading to the rational design of new materials that aim to mitigate detrimental vibrational effects through chemical modification and crystal engineering.^[^ ^42^, ^43^, ^44^, ^45^, ^46^ ^]^ This has corresponded to parallel advances in experimental and computational methods that enable directly capturing the nuclear motions responsible for such effects.^[^ ^43^, ^47^, ^48^, ^49^, ^50^ ^]^

Over the last decade, these efforts have accelerated and have resulted in a wave of research focused on understanding the specifics of the intermolecular interactions that dictate such dynamics. Specifically, recent efforts have highlighted that the so‐called “thermally activated dynamics” are in fact quantized low‐frequency vibrational modes, which tend to have frequencies in the terahertz (far‐infrared) region of the electromagnetic spectrum (0.1–10 THz, 3–330 cm^−1^, 0.3–40 meV, λ = 3.1 mm – 30.3 µm). Progress in the experimental acquisition of low‐frequency vibrational spectra, including terahertz time‐domain spectroscopy (THz‐TDS), low‐frequency Raman spectroscopy, and inelastic neutron scattering (INS), coupled with advances in high‐performance computing and *ab initio* quantum simulations, has enabled unprecedented insight into the exact coordinates that are responsible for reducing charge carrier mobility in OSCs.^[^ ^43^, ^47^, ^49^, ^50^, ^51^, ^52^, ^53^, ^54^, ^55^, ^56^, ^57^, ^58^ ^]^ Through these combined experimental and theoretical approaches, the field has made substantial progress in elucidating and addressing the challenges posed by thermally induced dynamic disorder in OSCs—providing the community with new tools and techniques for understanding such phenomenon with atomic‐level insight, paving the way for the next generation of OSC materials. Yet—despite these significant advances in understanding—much debate exists regarding the exact nature of the phonon modes that predominantly affect charge transport. Specifically, the literature contains two contrasting views: one proposing that a single or very few phonon modes primarily dictate charge transport,^[^ ^47^, ^59^ ^]^ enabling targeted phonon engineering, and the other suggesting that no individual phonon mode dominates,^[^ ^43^, ^60^ ^]^ thus complicating efforts toward targeted phonon engineering and leading to a rich area of future research.

Building on these insights, significant efforts are now focused on utilizing detailed knowledge of molecular and lattice dynamics to design new materials that minimize harmful vibrational modes in OSCs.^[^ ^43^, ^61^ ^]^ Substantial progress has been made in tackling the challenges posed by thermally induced dynamic disorder; however, some key obstacles remain. The first is the computational cost of accurately simulating the low‐frequency dynamics of OSCs (and molecular crystals in general), which can become prohibitive as the molecular size increases.^[^ ^62^ ^]^ The second is the difficulties associated with the *a priori* prediction of bulk crystal structures for materials that have not been experimentally obtained, which limits the computational search for new efficient OSCs.^[^ ^63^, ^64^, ^65^, ^66^, ^67^, ^68^ ^]^ Moreover, using current simulation methods, it is challenging to calculate non‐gamma point phonon modes due to the computational cost, limiting many studies to analyzing optical phonons—despite the obvious importance of phonon dispersion on the actual dynamics present within a material.^[^ ^43^, ^48^, ^69^, ^70^, ^71^, ^72^ ^]^ Finally, a major challenge lies in bridging the gap between molecular‐level phenomena and practical device engineering, as the relationship between meso‐scale effects—such as defects, grain boundaries, surface passivization, and so on—on the overall performance of OSC devices is complex and not yet fully resolved. To overcome these hurdles, ongoing advancements in computational methods and experimental techniques are essential, as is the need for interdisciplinary collaboration to translate these fundamental discoveries into real‐world applications.

As the field advances, the integration of theoretical models with experimental data will be key to unlocking the full potential of OSCs. Understanding how molecular dynamics influence charge mobility at the atomic level opens new avenues for material design. This review aims to highlight the most exciting advances over the last few years while providing a realistic assessment of current challenges so that the community is well‐equipped to develop high‐performance organic electronics that overcome many of the limitations facing the field.

## 2 Dynamics, Dynamic Disorder, and Transient Localization Theory

### 2.1 Atomic‐Level Description of Molecular and Crystalline Vibrational Dynamics

The literature on electron–phonon coupling in OSCs frequently refers to “thermally activated” dynamics.^[^ ^30^, ^35^, ^73^ ^]^ These dynamics originate from quantized vibrational modes thermally populated at ambient conditions. Given the interdisciplinary composition of the OSC research community, it is valuable to clarify these concepts, particularly to bridge understanding across subfields and standardize terminology for better coherence in interdisciplinary collaborations.

In crystalline materials, vibrational motions result primarily from interatomic or intermolecular interactions, typically described within the harmonic approximation. The simplest representation of a vibrational potential energy surface, *V*(*Q*), for a given normal‐mode coordinate *Q*_*i*_ is described by:

(1) $$V_{i}\left(Q_{i}\right)=\frac{1}{2}k_{i}Q_{i}^{2}$$

Here, *V*_*i*_(*Q*_*i*_) is the potential energy (in units of energy, e.g., Joules), *k*_*i*_ is the force constant with units of force per distance (e.g., newtons per meter (N m^−1^), and *Q*_*i*_ is the generalized coordinate (displacement from equilibrium) with units of length (e.g., meters, m).

Solving the quantum harmonic oscillator Schrödinger equation yields discrete vibrational energy levels:

(2) $$E_{i,\nu }=\left(\nu +\frac{1}{2}\right)\hbar \omega_{i},\;\omega_{i}=\sqrt{\frac{k_{i}}{M_{i}}}$$

where ν is the vibrational quantum number, ℏ is the reduced Planck's constant, ω_*i*_ is the angular frequency, and *M*_*i*_ is the reduced mass associated with the mode.

**Figure 2:**
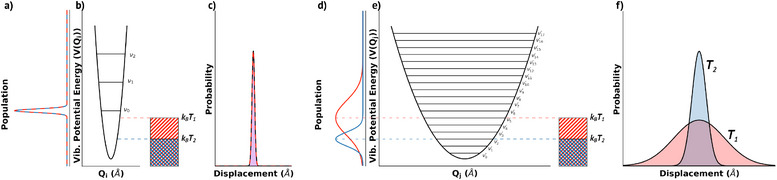
Schematic representation of two vibrational potentials with b) representing a stiff force constant and e) a soft force constant. Considering two temperatures, *T*_1_ and *T*_2_, the amount of thermal energy (*k*_*B*_ *T*) is shown, with the horizontal dashed lines representing the maximum potential energy possible for a classical thermal oscillator. Panels (a) and (d) show how changing temperature alters the thermal‐population of the vibrational states, with a) showing that the stiff potential only has the ground‐state populated at both temperatures, while d) shows that the soft potential exhibits a dramatic change in thermally populated states as temperature is decreased. This translates to the amount of thermally induced motion for each of these modes, with c) demonstrating that there is no change in the displacement amplitude for the two representative temperatures, while f) shows that the soft mode produces a large change in displacement amplitude as temperature is changed.

This fundamental relationship connects the stiffness of the vibrational potentials (quantified by *k*_*i*_) to vibrational frequencies (ω_*i*_) and their thermal populations at a given temperature. As illustrated in Figure 2, vibrational modes associated with stronger interactions (large *k*_*i*_) possess higher vibrational frequencies, and thus remain predominantly in the ground vibrational state at room temperature due to limited thermal excitation (Figure 2a,b). Conversely, softer interactions with lower frequencies exhibit significant thermal populations at ambient conditions, leading to larger amplitude displacements and thus more substantial impacts on intermolecular electronic coupling (Figure 2d,e).

In molecular crystals, the vibrational potentials arise from interactions between atoms and/or molecules, with the nature of the vibrational motion (i.e., the coordinates along which particles move) dictating the associated energies of the vibrational states.^[^ ^27^, ^62^ ^]^ Intramolecular vibrational modes, such as O–H stretching, involve displacement along covalent bonds and behave as “stiff” springs, with large force constants and high vibrational transition frequencies (e.g., 3400 cm^−1^ for O–H stretches). Variations in bond strength due to environmental factors are small, allowing for well‐defined transition frequencies for common functional groups (e.g., C─H and C═O stretches). Conversely, intermolecular interactions, weaker than covalent bonds, result in smaller force constants and thus lower‐energy vibrational frequencies. This distinction is critical for understanding dynamic disorder in OSCs.

**Figure 3:**
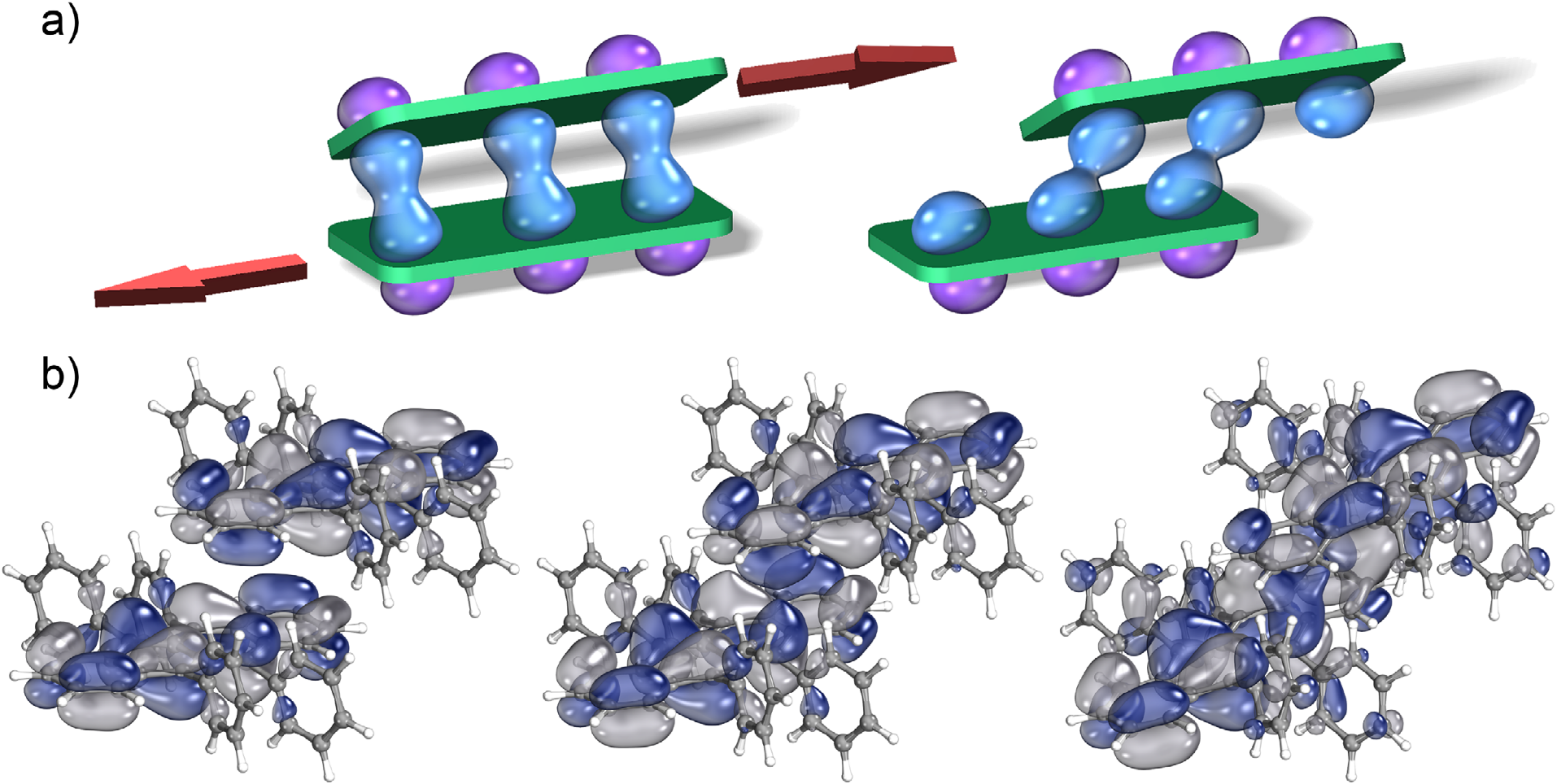
a) Cartoon representation of the modulation of intermolecular orbital overlap in a planar OSC‐like molecular dimer by long‐axis antisymmetric hindered translational motion, reproduced with permission from ref. [49] and b) the highest occupied molecular orbital in rubrene shown as a function of displacement along a predicted terahertz vibrational mode at 75 cm^−1^.

Molecules bound by van der Waals forces frequently exhibit intermolecular low‐frequency vibrational modes, often referred to as hindered translational and rotational modes.^[^ ^26^, ^27^ ^]^ These soft modes, involving entire molecules or large molecular fragments, typically lead to large displacements from equilibrium (0.1–0.5 Å) at ambient conditions due to their small transition frequencies and associated vibrational energies. These modes significantly modulate intermolecular orbital overlap and charge transfer integrals, contributing predominantly to dynamic disorder (see Figure 3).

Examples of this phenomenon are depicted in Figure 3. In OSCs, rigid‐molecule hindered translational modes along the molecular long axis have long been hypothesized^[^ ^30^, ^39^, ^73^, ^74^ ^]^ and later confirmed^[^ ^47^, ^49^, ^50^, ^75^ ^]^ as particularly detrimental to charge transport. As illustrated, antisymmetric translational motion substantially reduces intermolecular wavefunction overlap relative to the equilibrium structure. Figure 3b further demonstrates the modulation of the highest‐occupied molecular orbital (HOMO) overlap in rubrene, resulting from displacement along a vibrational mode at 76 cm^−1^.^[^ ^76^ ^]^ These examples are shown to illustrate the modulation of intermolecular dynamics on the electronic structure, as they are more clearly visualized here compared to other, more complex, examples.

The energetic origins promoting terahertz dynamics are inherently complex, stemming from entangled interatomic interactions and long‐range effects.^[^ ^27^, ^77^, ^78^ ^]^ Unlike mid‐infrared vibrations, low‐frequency modes exhibit no standard transition frequencies, as minor variations in molecular structure or crystal packing significantly alter vibrational potentials. Consequently, low‐frequency vibrational spectroscopy has become an essential tool for studying crystalline polymorphism.^[^ ^79^, ^80^, ^81^ ^]^ Terahertz vibrational potentials are so sensitive to intermolecular interactions that even subtle guest molecule orientation differences within framework materials drastically alter observed spectra.^[^ ^82^, ^83^ ^]^ This complexity presents both challenges and opportunities: while assigning and controlling these modes is difficult, tuning intermolecular interaction strengths provides a path to influence—and ultimately mitigate—the impact of thermally activated dynamics.^[^ ^43^ ^]^ This particular point is crucial for the rational design of OSCs, and it has recently been used successfully to generate a material with double‐digit mobility.^[^ ^43^ ^]^

### 2.2 Dynamic Disorder in OSCs

Although the exact nature of the charge transport mechanism in OSCs remains a topic of debate,^[^ ^74^, ^84^, ^85^ ^]^ it has been now clearly demonstrated that the fluctuations in intermolecular electronic interactions reduce the overall coherence of charge transport.^[^ ^86^, ^87^, ^88^ ^]^ Although early models of charge transport in OSCs relied on a purely incoherent hopping mechanism,^[^ ^89^, ^90^, ^91^ ^]^ described by Marcus theory, later observations revealed that this framework was insufficient for explaining the higher mobilities (often µ > 10 cm^2^ ·V^−1^ ·s^−1^) found in OSCs, along with their temperature dependence.^[^ ^86^ ^]^ In high‐mobility OSCs, fluctuations in intermolecular electronic interactions temporarily localize charge carriers and force them to hop between localized states, rather than allowing free movement through delocalized bands.^[^ ^87^, ^92^ ^]^ This realization led to the development of the transient localization theory (TLT), introduced by Fratini et al.,^[^ ^39^, ^41^ ^]^ which more accurately captures the dynamic behavior of charge carriers in OSCs.

Throughout this review, we focus on understanding the fundamental, atomistic origins of charge transport in OSCs. Specifically, our analysis is centered on describing the pristine, intrinsic transport properties of ideal crystalline materials, as governed by phonon modes, lattice dynamics, and electron–phonon interactions. We do not explicitly consider extrinsic or mesoscopic effects such as deposition methods, grain boundaries, impurities, or surface/interface defects, which are well known to influence real‐device performance.^[^ ^93^, ^94^, ^95^, ^96^, ^97^, ^98^, ^99^, ^100^ ^]^

#### 2.2.1 Transient Localization Theory

Transient localization theory provides a detailed framework for taking into account dynamic disorder to determine charge carrier mobility.^[^ ^39^ ^]^ This theory accounts for the fluctuations in intermolecular transfer integrals, which are the probabilities of charge transfer between neighboring molecules. These fluctuations are caused by thermally activated phonon modes, which generates a dynamic electronic landscape that transiently localizes the charge carrier energy levels.

In OSCs, charge carriers experience transient localization over a length scale *L*_τ_, determined by the fluctuation time τ, which is inversely proportional to the typical molecular vibration frequency ω_0_. The charge carrier mobility can be expressed analytically using a relaxation time argument, incorporating quantum localization corrections. The formula for charge mobility in the TLT models is:

(3) $$\mu =\frac{e}{k_{B}T}\frac{L_{\tau }^{2}}{\tau }$$

The critical quantity to consider here is *L*_τ_, which is computed by utilizing dynamic disorder–deduced coupling values.^[^ ^40^ ^]^

Although there is significant work underway to uncover tools to calculate accurate mobilities from simulated data, TLT represents one approach that is being widely utilized, yet others are currently being investigated. However, this is outside of the scope of this current review, and therefore, we direct the reader elsewhere for further reading.^[^ ^101^, ^102^, ^103^, ^104^ ^]^

#### 2.2.2 Globally Resolving Dynamic Disorder Experimentally and Theoretically

Early efforts to reduce dynamic disorder took an aggregate approach of molecular dynamics to understand how thermalized dynamics influence intermolecular electronic coupling and thus how dynamics modulate charge transfer in OSCs.^[^ ^37^, ^105^, ^106^, ^107^, ^108^, ^109^ ^]^ In such studies, it was common to utilize molecular dynamics (MD) simulations, which provide a global view of molecular motions without mode‐specific insight. For example, Troisi and coworkers studied functionalized pentacene derivatives using classical MD simulations, followed by computing the electronic structure for dimers extracted from the solid at standard time‐intervals (in this case, 0.1 ps).^[^ ^37^ ^]^ This procedure provides a time‐averaged description of the fluctuation in the electronic structure and intermolecular orbital couplings, and is effective for understanding how thermally activated vibrational motions play a role in charge transfer in organic electronics, and this approach has been replicated by many other works in later studies.^[^ ^29^, ^31^, ^109^ ^]^It is important to note that utilizing MD simulations for these efforts do provide a benefit in that they are not limited to gamma‐point phonon modes, and thus provide a more “physical” interpretation of the link between dynamics and electronic coherence.

However, the downside to using MD simulations to analyze dynamic disorder in OSCs is that they do not readily provide mode‐specific insight—namely, they are not able to directly quantify how individual vibrational coordinates contribute to dynamic disorder without significant post‐processing of the MD‐generated trajectories.^[^ ^110^ ^]^ Although the complete impact of nuclear motions on dynamic electronic disorder can certainly be uncovered from such simulations, the lack of mode‐resolved insight does not provide an opportunity to understand the precise origin of the particularly detrimental effects without additional analyses, which is essential for utilizing the computed information to rationally design new materials.

This approach has also been taken in complementary experimental studies. In the early 2010s, the synthesis and characterization of some high‐performance OSC cores that were decorated with pendant alkyl chains revealed that such functionaliztion dramatically improved charge carrier mobility.^[^ ^111^, ^112^ ^]^ The hypothesis developed to explain such phenomena suggested that alkylation served to “lock” the aromatic cores in their sites, leading to the suppression of thermally activated dynamics and thereby improving mobility.^[^ ^42^, ^111^, ^113^, ^114^ ^]^ In 2016, Illig et al. provided an experimental evaluation of the amplitude of thermal lattice fluctuations in multiple OSCs that included such chemical modifications through the analysis of diffuse scattering features in transmission electron diffraction experiments.^[^ ^73^ ^]^ There, the authors utilized electron diffraction to study small‐molecule OSCs like BTBT and C8‐BTBT. By analyzing the ellipsoial nature of the Bragg reflections, the amount of thermally induced motion was correlated to the trends in mobilities across samples. This revealed that large‐amplitude thermal vibrations, particularly along the long axis of the conjugated cores, are present in these materials, and the resulting findings showed correlations between molecular functionalization, the spatial magnitude of these large‐amplitude motions, and experimentally determined mobilities, particularly highlighting that reduction of motion in certain crystallographic directions yielded higher charge carrier mobility. This work suggested that materials where side chains are attached to the long axis of the conjugated core, such as C8‐BTBT, exhibited smaller nuclear displacements compared to the unalkylated base aromatic units. This pointed to the hypothesis that the suppression of long‐axis vibrations are responsible for most‐strongly modulating intermolecular charge transfer. However, these experiments again do not provide mode‐specific insight, as the analysis of the diffuse scattering provides a time‐ and spatial‐average of all of the atomic positions within the volume of the sample irradiated with the electron beam. Thus, they provide a holistic average picture of the degree of nuclear motions, without any mode‐resolved information, i.e., which coordinates are the ones that are most‐strongly influenced by dynamic disorder. In that sense, they are similar to the computational studies using MD simulations.

Thus, although these studies strongly support that dynamic disorder is crucial for understanding charge transport in OSCs, they were only indirectly capable of providing atomic‐level insight into the precise coordinates that dictate such effects.

#### 2.2.3 Mode‐Resolved Description of Dynamic Disorder

In recent years, these questions have been addressed using a combination of experimental and theoretical methods.^[^ ^47^, ^50^ ^]^ On the experimental front, studies that have utilized experimental low‐frequency vibrational spectroscopy measurements, including terahertz time‐domain spectroscopy, low‐frequency Raman spectroscopy, and inelastic neutron scattering, are able to directly measure the vibrational mode frequencies for the same dynamics that are most strongly influenced by temperature.^[^ ^47^, ^50^, ^55^, ^56^ ^]^ On the theoretical front, there are two, nearly similar, yet distinct, methods that have been proposed for capturing mode‐resolved information in this regard.^[^ ^30^, ^31^, ^115^, ^116^, ^117^ ^]^ Ultimately, the key quantity that is required involves understanding how the intermolecular charge transfer integral, *J*, is influenced by a vibrational coordinate,

(4) $$\beta_{mn,i}={\left(\frac{\partial J_{mn}}{\partial Q_{i}}\right)}_{0}$$

where β_*mn*, *i*_ is the derivative of the charge transfer integral (*J*, units of energy) with respect to vibrational coordinate (*Q*, with units of length) for a pair of molecules with indices *m*, *n* along a particular vibrational mode *i*—which ultimately produces units of energy per distance (see illustrations of interacting molecules and the notation of the associated transfer integrals between molecular pairs in Figure 1). This treatment generates the fluctuation in the intermolecular charge transport integral on a normalized basis, and does not take into account thermally activated displacements. In order to achieve this, the variance (or spread) of the thermal amplitudes of vibrational modes are calculated via the following expression,

(5) $$\sigma_{i}^{Q}=\sqrt{\frac{k_{B}T}{M_{i}\omega_{i}^{2}}}$$

where $\sigma_{i}^{Q}$ is the thermal amplitude for mode *i* with a frequency of ω at temperature *T*, and *k*_*B*_ is the Boltzmann constant. In order to obtain the mode‐resolved value of dynamic disorder (σ_*mn*, *i*_) at a particular temperature (taking into account the amplitude of thermally‐excited dynamics), the product of these two quantities are taken

(6) $$\sigma_{mn,i}=\beta_{mn,i}\sigma_{i}^{Q}$$

with σ_*mn*, *i*_ representing the dynamic disorder of dimer pair *mn* for mode *i* at a particular temperature.

In one practical approach to determining these quantities, Schweicher et al. proposed that the transfer integral derivatives, β_*mn*, *i*_, are determined on a mode‐by‐mode basis, i.e., the system is displaced individually along each vibrational mode and β_*mn*, *i*_ redetermined for each one.^[^ ^47^ ^]^ That work also showcases how the results of this type of analysis are based on ideal single crystalline devices, as theoretical predictions of mobility based on phonon‐limited transport models consistently overestimate experimental.^[^ ^47^ ^]^ This is expected, since the calculations capture only the fundamental, phonon‐driven contributions to charge scattering and ignore the additional resistive effects from structural defects and mesoscopic disorder present in real devices.^[^ ^95^, ^96^, ^97^, ^98^, ^99^, ^100^, ^101^, ^118^ ^]^ Thus, the theoretical predictions should be interpreted as an upper bound on mobility in the pristine crystalline phase.

An alternative approach from Harrelson et al. instead involves computing a spatially‐resolved map of the transfer integral itself, ultimately determining its gradient (∇*J*).^[^ ^50^ ^]^ Then, instead of re‐determining β_*mn*, *i*_ numerically on a mode‐by‐mode basis, the vibrational coordinates of a mode *i* are projected onto ∇*J*_*mn*_,

(7) $$g_{mn,i}=\nabla J_{mn}\cdot Q_{i}.$$

The overall variance on atoms is given by the summation of *g*_*mn*, *i*_ weighted by the frequency of each mode, ω_*i*_.

(8) $$\sigma_{\mathrm{mn}}^{2}=\frac{1}{N_{q}}\sum_{i}\frac{{\left(g_{\mathrm{mn},i}\right)}^{2}}{2}\coth\left(\frac{\hbar \omega_{i}}{2k_{B}T}\right).$$

**Figure 4:**
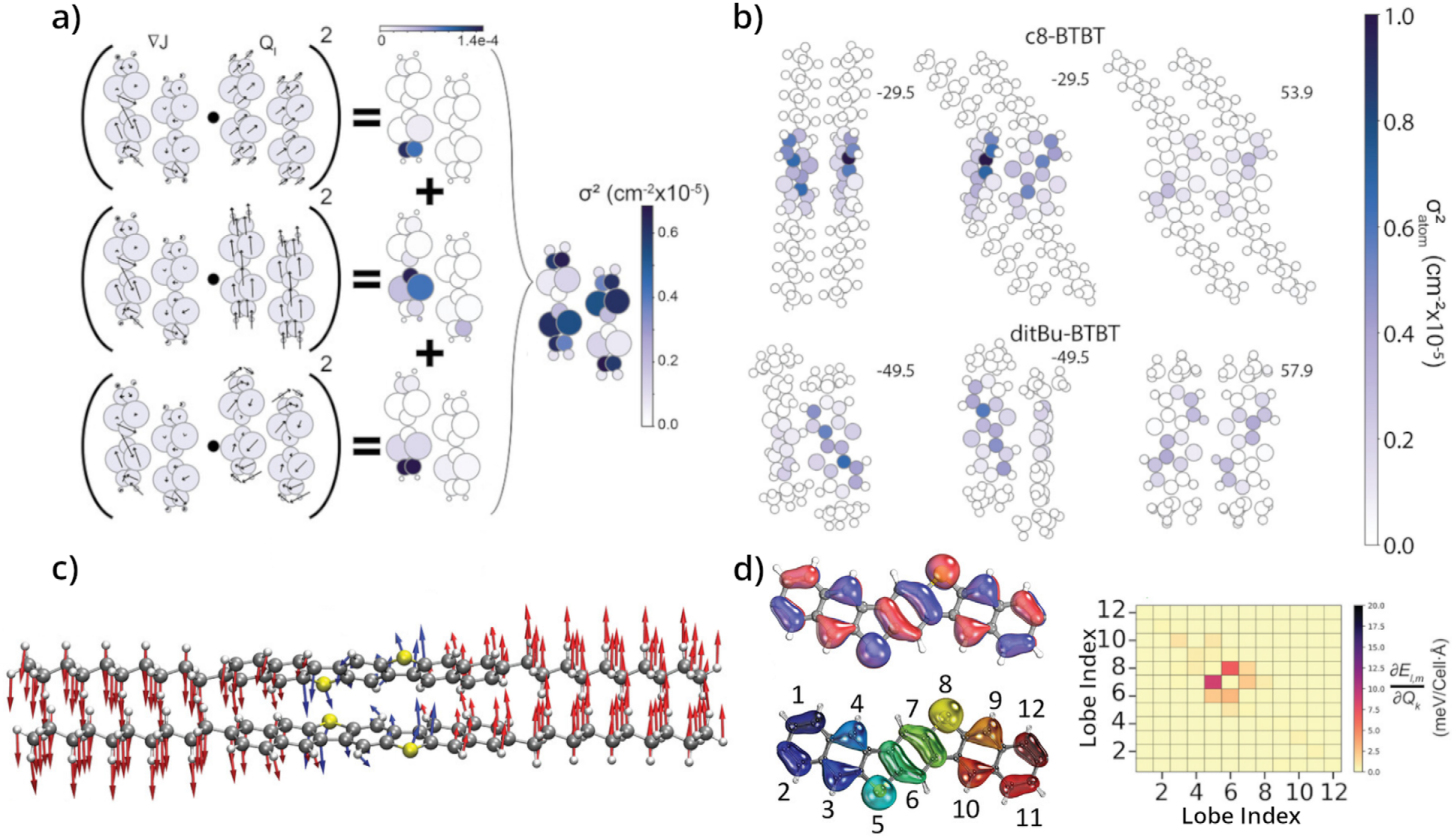
Representations of Dettmann et al.^[^ ^43^ ^]^ method via the gradient of the transfer integral Δ*J*, panels (a) and (b), and the mode‐by‐mode approach from Schweicher et al.,^[^ ^47^ ^]^ panels (c) and (d). a) Schematic representation of the gradient approach. For each mode *Q*_*i*_, the inner product Δ*J* · *Q*_*i*_ is evaluated and squared, leading to the visualization of atomic contributions for each electron–phonon coupling heatmaps in the central panel of (a). Individual contributions are then summed up for the variance σ^2^ for each atom (see Equations 7 and 8), that is also visualized as a heatmap. b) Heatmap for σ^2^ distribution on each atom in two alkylated BTBT derivatives, C8‐BTBT and ditBu‐BTBT. c) Schematic representation of one detrimental mode in C10‐DNBDT‐NW, reported by Banks et al.^[^ ^49^ ^]^ This mode involves a rotation about the molecular short‐axis and was found to contribute approximately 27% to the total dynamic disorder. d) HOMO electron density with discrete in‐phase lobes numbers, and the associated energetic modulation for each pair of neighboring orbital–lobe interactions obtained using the crystal orbital Hamiltonian population method. Reproduced with permission from Ref. [43] ‐ (a) and (b); and Ref. [49] ‐ (c) and (d).

In Equation 8, *N*_*q*_ accounts for the number of points in the reciprocal space. The procedure outlined here is illustrated in Figure 4a, and examples of its application to two OSCs are shown in Figure 4b. Although these two approaches generate the same quantities, the latter method has the benefit of reducing the number of required calculations, and if ∇*J* is determined to a high‐enough spatial resolution, likely provides a more robust and straightforward means to capturing dynamic disorder with full mode‐resolved resolution.

Importantly, although the discussion above largely assumes harmonic lattice dynamics, organic crystals are well‐known to exhibit significant anharmonicity due to their weak intermolecular forces.^[^ ^76^, ^119^, ^120^, ^121^ ^]^ Anharmonic lattice vibrations lead to effects such as temperature‐dependent phonon frequencies, mode coupling, and non‐linear modulations of charge transport integrals, all of which complicate the electron–phonon interaction landscape. Recent work by Asher et al.^[^ ^54^ ^]^ has shown that chemical modifications to OSCs can suppress these anharmonic effects, leading to more coherent phonon spectra and improved charge mobility. This suggests that understanding and ultimately controlling vibrational anharmonicity could provide an additional lever for rational phonon engineering.

## 3 Designing OSCs with Dynamics in Mind

The discovery that thermally activated dynamics, particularly low‐frequency vibrational modes, play a critical role in limiting charge transport in OSCs has fundamentally shifted the approach to material design. Building on insights from both experimental and theoretical studies, new strategies for the rational design of OSCs have emerged, with a focus on minimizing the detrimental effects of dynamic disorder. Early efforts in the field were largely heuristic, focused on enhancing molecular rigidity to reduce molecular vibrations. However, recent advancements in understanding the specific vibrational modes that affect charge transport have paved the way for more precise design principles.^[^ ^47^, ^49^, ^87^, ^122^, ^123^ ^]^

### 3.1 Early Approaches to Holistically Reduce Dynamic Disorder

Initial strategies to mitigate dynamic disorder involved attempts to suppress large‐amplitude molecular vibrations of the regions of the molecular units believed to be involved in the charge transport processes.^[^ ^113^, ^114^, ^124^ ^]^ However, prior to the introduction of dynamic considerations, other factors took precedence. Originally some of the high‐mobility OSCs discovered in the 2000s, such as TIPS‐pentacene and other functionalized acenes, were not originally designed with the mitigation of dynamics in mind.^[^ ^125^, ^126^, ^127^ ^]^ Instead, the motivation for those early efforts were to add functional groups that would improve to solubility of the materials, with the additional aim that the side‐chains would promote a co‐facial arrangement of the aromatic acene cores, which would maximize overlap of the π‐orbitals and thus ideally improve charge carrier mobility.^[^ ^125^, ^126^, ^127^ ^]^ This is similarly the case for the functionalized derivatives of the well‐known thiophene‐based OSC, BTBT, which exhibited promising charge carrier mobilities.^[^ ^128^ ^]^ However, in that work, there is brief mention of the interactions between neighboring alkyl chains increases the interaction strength between adjacent molecules—while initially discussed through the lens of favorable supramolecular assembly, planted the seeds for the later focus on reducing dynamics.

A subsequent study, from the same group that reported the alkylated BTBT derivatives, reported the synthesis and characterization of alkylated DNTT derivatives, which again achieved rather high mobilities of around 8 cm^2^ ·V^−1^ ·s^−1^.^[^ ^112^ ^]^ Although creating more soluble materials was indeed a focus, that report also included mention that alkylation served to pack the aromatic molecular core more tightly through increased van der Waals interactions along the length of the side‐chain, which was attributed to the so‐called “molecular fastener effect.”^[^ ^129^ ^]^ From here, as the field began to appreciate the critical role that lattice dynamics play on charge carrier mobility in OSCs, the hypothesis that the increased interactions afforded to the system by side‐chain functionalization reduced detrimental motions, along with the experimental observation of increasing mobility upon alkylation, was increasingly referenced.

It is clear that a shift in the community began to occur in the early 2010s. For example, in 2013, Okamoto et al. reported the synthesis and characterization of a new class of OSCs based on a “V‐shaped” design, which was motivated by a desire to create materials with good solution processability, high mobility, and high thermal durability in order to survive device fabrication—as many of the high‐performance OSCs up until that point melted at temperatures below standard deposition conditions.^[^ ^113^ ^]^ A year later, the same group reported a different, but related, molecular design based on a “N‐shaped” core.^[^ ^114^ ^]^ Interestingly, that work was largely motivated by reducing thermal dynamics (i.e., dynamic disorder), based on studies in the preceding years from Takeya^[^ ^32^ ^]^ and Troisi,^[^ ^106^ ^]^ respectively. From there, the consideration of lattice dynamics on charge transport has been increasingly integrated into experimental design strategies. Another example is the study conducted by Thomas et al.^[^ ^130^ ^]^ on a class of OSCs composed of 2D organic materials known as a covalent organic framework. In this study, the authors reported a series of OSCs with theoretical charge carrier mobilities > 66 cm^2^ ·V^−1^ ·s^−1^. In that work, the authors explicitly attempt to take into account vibrational motions—however, they do not perform periodic simulations, and restrict their calculations to considering the vibrational modes involving only a dimer pair—likely obscuring the true nature of the crystalline dynamic disorder. However, this work showcases how understanding vibrational dynamics can provide insight into OSC performance.

### 3.2 Utilizing Mode‐Resolved Insight to Design OSCs

Efforts to design OSCs to mitigate detrimental thermal dynamics thus began prior to the ability to fully‐resolve, on a mode‐by‐mode basis, the role of vibrational motions on charge transport.^[^ ^124^, ^131^ ^]^ This resulted in some assumptions and general strategies that, while intuitive, were not fundamentally correct as they were based on a classical view of dynamics, instead of relying on specific insight related to quantized vibrational dynamics. For example, there were a number of studies in the mid‐2010s that reported attempts to “lock” the semiconducting cores based on chemical modification. Yet, no complementary studies were performed related to the degree of suppression, the frequencies of the various vibrational modes, the relationship between particular dynamics and dynamic disorder, and so on.^[^ ^132^, ^133^, ^134^, ^135^, ^136^ ^]^ Thus, although the materials generated based on these assumptions are certainly high‐performance and likely do exhibit reduced motions along some coordinates, such a simplistic description obscures the precise origins of the improved mobilities, and therefore, the ability to leverage fundamental factors for “true” rational design. Without such insight, pitfalls can, and have, been encountered by the OSC community.

Such pitfalls were only uncovered once a mode‐resolved picture came into focus. Work in 2019 from Schweicher et al. studied a class of high‐performance OSCs, many of the same studied in 2016 by Illig,^[^ ^73^ ^]^ using a combination of experimental terahertz time‐domain spectroscopy, inelastic neutron scattering spectroscopy, and solid‐state density functional theory simulations.^[^ ^47^ ^]^ The motivation of that study revolved around uncovering the origins of the increased mobility of alkylated derivatives of common OSCs, including BTBT and DNTT. Interestingly, the results of those efforts showed that, counter to the prevailing understanding at the time (which was backed up by previous works), alkylation does not alter the frequency of the long‐axis sliding vibrational mode—and by extension, the magnitude of the thermally‐activated displacement—contrary to what was reported previously.^[^ ^35^, ^73^ ^]^ Instead, alkylation served to increase the frequency of torsional dynamics of the fused core, decreasing the detrimental effect of these motions and thereby minimizing the impact of such modes on dynamic disorder. One powerful outcome from that study was that in the alkylated derivatives, the vast majority of the detrimental electron–phonon coupling energy arose from just a single mode—the same long‐axis sliding mode that was believed to be minimized by alkylation.

Some additional pioneering work to resolve mode‐specific electron–phonon couplings in OSCs came from Harrelson et al., who introduced techniques that integrated *ab initio* simulations with inelastic neutron scattering.^[^ ^50^ ^]^ Their mode‐resolved approach enabled the authors to quantify the impact of specific vibrational modes on dynamic disorder by calculating the gradient of the charge transfer integral (∇*J*), followed by applying ∇*J* to the normal mode coordinates, *Q* (see Equation 7). In one early work, Harrelson et al. showcased that 67%–97% of the detrimental electron–phonon coupling in a large series of OSCs arose from low‐frequency vibrational modes, somewhat contradicting the hypothesis that it is solely low‐frequency vibrations that drive dynamic disorder.^[^ ^47^ ^]^ Furthermore, that work also highlighted the importance of considering the entire Brillouin zone, instead of gamma‐point only phonons, for fully‐capturing dynamic disorder in OSCs.

Follow‐up efforts from Banks et al. and Dettmann et al. utilized different mode‐resolved approaches to not only understand the electron–phonon coupling across a wider class of materials, but also to attempt to include the ability to pinpoint particular atomic origins of such effects.^[^ ^43^, ^49^ ^]^ Both of these studies revealed interesting phenomena that run slightly counter to what had been reported previously. In the latter study, Dettmann projected the combined contribution of all vibrational modes on the total electron–phonon coupling onto individual atoms in the system, enabling the clear visualization of the origins of detrimental effects, as shown in the heatmap in Figure 4b for two BTBT alkylated derivatives. One surprising outcome was the outsized role of a high‐frequency vibrational mode at around 1600 cm^−1^ in BTBT and its alkylated derivatives, which was significantly detrimental to charge transport. Since this vibrational involves a stretching of the aromatic C─C bonds, which promotes a breathing‐type motion of the entire ring system, it is likely that the origin of this effect is related to the modulation of the π‐electron wavefunction, which significantly alters the intermolecular transfer integral. Interestingly, while the low‐frequency phonons were strongly‐influenced by dispersion, the high‐frequency mode was not—likely due to the more localized nature of the mid‐IR motion. Similarly, Banks achieved new insights by evaluating the change orbital overlap as a function of mode using the crystal orbital Hamiltonian population analysis originally devised by Roald Hoffmann and others^[^ ^137^, ^138^, ^139^ ^]^ (see Figure 4d).^[^ ^49^ ^]^ That work unveiled that even when attempting to reduce dynamics overall, there can still be some unintended motions that severely contribute to dynamic disorder. For example, C10‐DNBDT‐NW, which has reported mobilities around 16 cm^2^ ·V^−1^ ·s^−1^,^[^ ^114^ ^]^ exhibited a (now expected) long‐axis sliding vibrational mode with a frequency of 22 cm^−1^ that had contributed ca. 36% to the total energetic disorder at room temperature.^[^ ^49^ ^]^ Surprisingly, there was a second, nearly‐as‐detrimental motion (contributing ca. 27% to the total disorder), that involved a rotation about the molecular short‐axis (see Figure 4c). By carefully investigating the contributions of various atomic orbitals to the total modulation of the intermolecular overlap integral (Figure 4d), it was shown that because of the extremely large and efficient overlap of the sulfur atom–π interaction between adjacent molecules, the rotational motion severely disrupted that overlap, leading to the observed phenomenon.

Such an analysis can also help to provide insight into what sort of functionalization is best avoided from a dynamic standpoint—and subsequently, how to mitigate the effect through further molecular modification. One example of this was an investigation into a series of materials that contained a fused central aromatic ring system functionalized with peripheral thiophene‐based side chains.^[^ ^122^ ^]^ In that work, the co‐planar arrangement of the thiophene side chain promoted extended conjugation from the core to the side group. However, the thiophene moieties were prone to undergo low‐frequency hindered torsional motions that severely increased energetic disorder in such systems, rendering them nearly useless as OSC materials. A related study from Maleki and coworkers further expands on this point, attempting to stiffen the core‐thiophene dihedral angle rotational potential through the introduction of fluorine atoms that can interact with the side‐chain thiophene.^[^ ^44^ ^]^ There, this stiffened interaction yielded an impressive order‐of‐magnitude increase in mobility, from 1.4 cm^2^ ·V^−1^ ·s^−1^in the non‐fluorinated derivative, to 13.2 cm^2^ ·V^−1^ ·s^−1^in the fluorinated system. This result ultimately highlights that there are multiple avenues available for controlling detrimental interactions through weak non‐covalent interactions, and encourages future studies in this direction.

Although numerous design strategies based on mode‐resolved analyses have been proposed, distinguishing genuinely effective methods from coincidental outcomes remains challenging. Indeed, some reported enhancements in charge‐carrier mobility attributed to vibrational modifications might be isolated instances rather than broadly applicable solutions. For example, while certain substitutions in BTBT derivatives have effectively suppressed detrimental low‐frequency modes, attempts to generalize this strategy to other molecular frameworks such as acenes or oligothiophenes have sometimes yielded inconsistent or negligible results, underscoring the system‐specific nature of phonon engineering approaches. Therefore, careful consideration and comprehensive validation through systematic comparative studies across diverse molecular platforms are essential to establish general principles for vibrational mode manipulation. Such rigorous evaluation will allow the OSC community to better discern strategies with broad applicability from those limited by specific molecular or crystalline contexts, ultimately guiding the rational design of future high‐performance organic semiconductors.

## 4 Future Challenges and Trends in the Design of High‐Performance OSCs

The previous section serves to highlight the difficulty in engineering OSCs with dynamics in mind, and despite many claims in the literature to the contrary, the lack of a general set of design rules that integrate all of these various considerations.^[^ ^43^, ^45^, ^49^, ^57^, ^101^ ^]^ For example, future experimental and theoretical studies must explicitly address the ongoing debate—whether single or multiple phonon modes dictate transport—to validate practical strategies for phonon engineering in OSCs. Yet, despite these outstanding questions, there have been major steps forward in recent years as the fundamental link between vibrational dynamics and charge transport is further uncovered.

A key limitation in current phonon‐engineering approaches is the difficulty in generalizing insights across diverse OSC systems. Indeed, many of the successful examples highlighted thus far—such as the engineering of resilient molecular frameworks by Okamoto and colleagues—represent individual, system‐specific cases rather than broadly applicable strategies. This inherent limitation stems from two factors, the first is the difficulty in generalizing information across diverse chemical systems, and the second is the substantial computational cost and complexity associated with accurately simulating phonons, electron–phonon coupling, and dynamic disorder across a wide range of molecular crystals. For example, there are currently outstanding questions over the influence of various phonon mode types and frequencies on dynamic disorder in OSCs. As discussed in the report from Dettmann et al.,^[^ ^43^ ^]^ and some others,^[^ ^60^ ^]^ the role of high‐frequency localized dynamics can play an important role on electron‐phonon coupling, which is seemingly at odds with other studies that promote the notion that it is a single low‐frequency phonon mode that causes the most detrimental effects.^[^ ^47^, ^59^, ^140^ ^]^ Yet, it is important to stress that these two “viewpoints” are not as contrasting as it might seem, as in the majority of the reported examples, there are OSC materials that have their electron–phonon coupling driven by multiple vibrations spanning different frequency ranges,^[^ ^60^ ^]^ especially those that are comprised of only the molecular “core” such as BTBT (compared to, for example, alkylated derivatives such as C8‐BTBT). Thus, in practice, such efforts to reduce the impact of charge carrier dynamics to a single variable (here, a single vibrational mode) might be misguided, and a more holistic approach taking into account all possible dynamics, is more valuable. Yet, in both cases, it is important to stress that an understanding of vibrational dynamics is key to future rational design, regardless of what is discovered in the fundamental investigation.

These difficulties and questions have consequently resulted in phonon‐related insights often emerging *a posteriori* rather than from predictive, rational design. Nevertheless, the rapid advancement of computational tools, particularly the incorporation of machine learning and artificial intelligence methods into computational workflows, offers promising avenues to overcome these limitations. For example, recent studies have shown that such approaches can effectively predict new targets for experimental crystallization through combined approaches.^[^ ^141^, ^142^, ^143^, ^144^ ^]^ Such methods could drastically reduce computational expense, enabling the predictive exploration of phononic landscapes across diverse molecular systems. This would transform phonon engineering from a retrospective analytical tool into a truly predictive component of rational OSC design. Therefore, concerted efforts are needed to further develop and integrate computationally efficient predictive approaches that could generalize phonon‐engineering principles, ultimately enabling the systematic design of OSCs with optimized dynamic and electronic properties.

**Figure 5:**
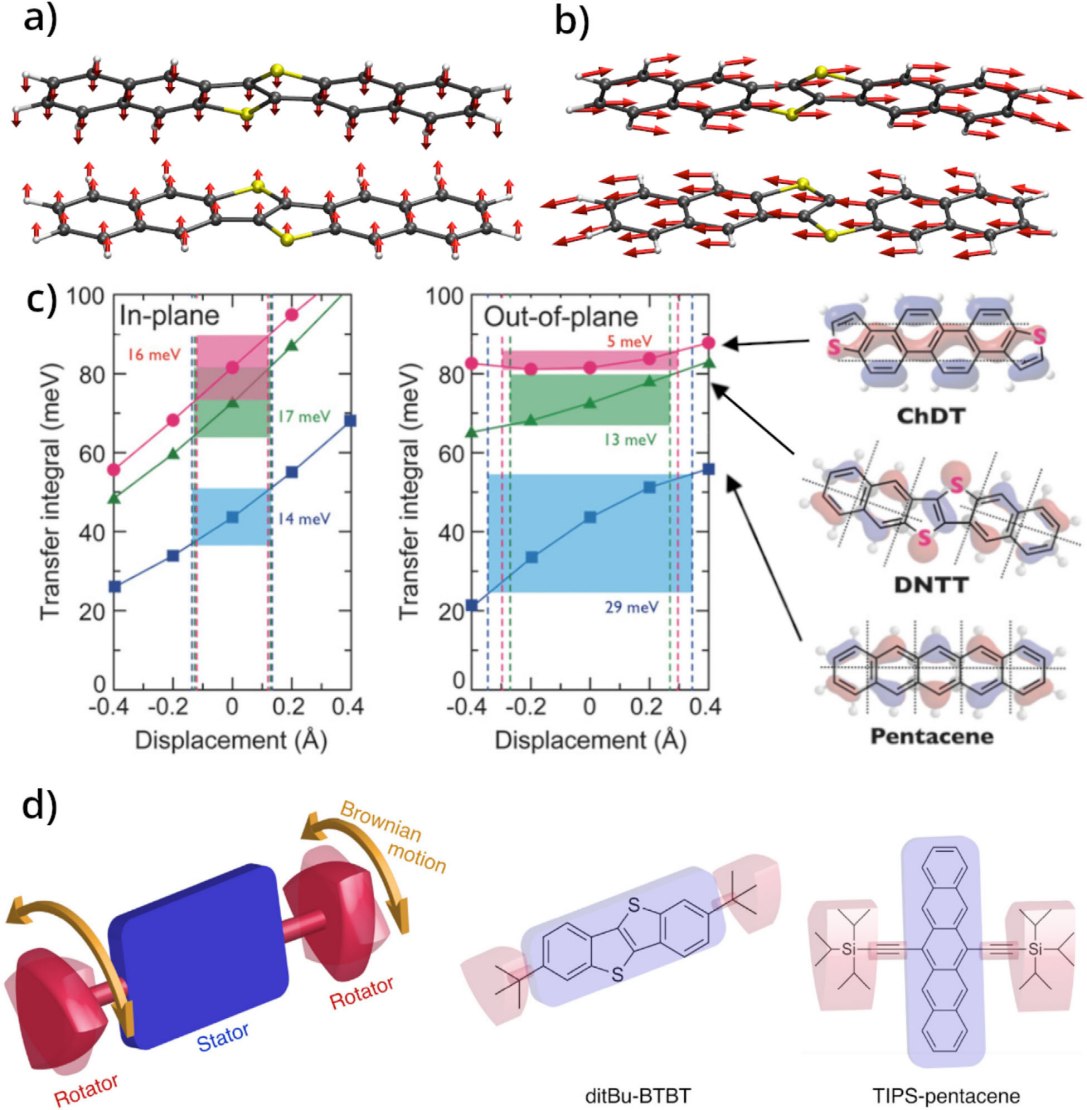
In‐plane a) and out‐of‐plane b) displacements discussed in Ref. [145], generated using the vibrational data reported by Schweicher et al.,^[^ ^47^ ^]^ and represent a zone‐boundary acoustic mode a) and the long‐axis sliding “killer” mode b) in DNTT. c) The transfer integral *J* for three OSCs, ChDT, DNTT, and pentacene calculated as a function of displacement from equilibrium for the two mode‐types shown in (a) and (b). d) Schematic representation of an amphidynamic molecule, composed of a rigid stator and a dynamic rotator, and examples of amphidynamic OSCs– ditBu‐BTBT and TIPS‐pentacene. Reproduced and modified with permission from Ref. [145] ‐ (c) and Ref. [147] ‐ (d).

The overarching consideration involves the need to balance favorable and large electronic overlap of the frontier orbitals, while simultaneously reducing dynamics as much as possible through a stiffening of the most critical intermolecular coordinates—which can now be understood by performing mode‐resolved analyses. One example where this has proven to be highly successful is the idea from Okamoto and colleagues involving the engineering of molecular orbitals to be resistant to displacive dynamics.^[^ ^145^, ^146^ ^]^ In recent work, Okamoto et al.^[^ ^146^ ^]^ proposed a new OSC, ChDT, which exhibits a zigzag shape and features an elongated π‐electronic core that was designed strategically to be resilient to long‐axis sliding vibrational modes that aim to overcome fluctuations in *J* and thus enhance charge carrier mobility. This work also highlights the importance of considering phonon dispersion, as the in‐plane motion they showcase does not correspond to an optical phonon, and instead represents an acoustic mode at the Brillouin zone boundary (see Figure 5a,c). This has been further exemplified by a recent review from Nematiaram and Troisi,^[^ ^45^ ^]^ where a screening of over 5000 OSCs revealed that one of the most likely markers of a high‐performance material was a charge transfer integral that had a small gradient with respect to nuclear displacement. Thus, it is crucial that steps be taken to understand both electronic structure and intermolecular potential energy surfaces in order to truly rationally‐design new materials.

One key challenge to OSC design is controlling not only molecular properties, but also bulk non‐covalent interactions, such as π–π interactions, hydrogen bonding, and halogen bonding, which dictate the crystal packing and ultimately influence charge transport properties.^[^ ^123^, ^148^, ^149^, ^150^ ^]^ Although much attention has been given to optimizing these interactions to control the supramolecular architecture of organic functional solids,^[^ ^148^ ^]^ the role of these weak forces in modulating the inherent lattice dynamics of crystalline materials, and specifically OSCs, remains underexplored. There are a few examples where such an approach has been undertaken, for example Roche et al. included hydroxyl groups and hydrolyzable and crosslinkable triethoxysilyl terminations on the alkyl side chains of BTBT to attempt to increase interlayer interactions and potentially promote interlayer‐crosslinking in order to stiffen the individual molecular units.^[^ ^151^ ^]^ In an alternative scenario, there are an increasing number of studies that are taking advantage of particular dynamics to promote properties, for example a recent report from Catalano and coworkers have shown that by designing amphidynamic organic crystals, illustrated in Figure 5d, which are built from both a rigid and highly mobile molecules and/or molecular fragments, a number of interesting macroscopic phenomena can be enabled.^[^ ^152^, ^153^ ^]^ In such crystalline materials, tailored atomic—and corresponding bulk—dynamics can be exploited for a broad range of functions and applications, such as gas storage, tunable photoluminescence properties, switchable dielectrics, and molecular sensing.^[^ ^108^, ^150^ ^]^ Thus, such materials (many of which are OSCs) can be implemented in practical settings that require tailored properties simply by regulating the lattice dynamics that occur.

In this sense, there is a profound opportunity utilizing computational methods to identify new materials in silico prior to undertaking experimental approaches. Ideally, these computational studies would take both crystalline structure, electronic structure, and the associated lattice dynamics into account simultaneously. Yet, although it is straightforward to suggest such an approach, it is much more challenging in practice due to the available computational techniques that enable accessing each of those considerations.

Over the last two decades, the *a priori* prediction of molecular crystal structures has evolved into a robust area of research that has yielded significant successes.^[^ ^63^, ^64^, ^65^ ^]^ Crystal structure prediction (CSP) involves the generation of a large number of bulk structures that are then ranked based on calculated energies—with the calculation engine dictating both accuracy and computational cost.^[^ ^66^, ^67^, ^68^ ^]^ Although promising, there are still many areas for improvement for CSP studies, including performing an energy analysis that balances computational cost and accuracy,^[^ ^154^ ^]^ the handling of molecular structures that have various conformations, and discerning between various metastable (and thus accessible) polymorphic forms.^[^ ^155^, ^156^ ^]^ Despite these challenges, CSP has already proven to be extremely useful for OSC design, as demonstrated by the discovery of a new ultrahigh mobility OSC from Takimiya and colleagues.^[^ ^46^, ^157^ ^]^ The inclusion of more studies that incorporate computational CSP into their OSC design strategy, potentially making use of machine learning methods (as demonstrated in a few recent examples),^[^ ^141^, ^142^, ^143^, ^144^ ^]^ is all but certain to pay dividends in the discovery of high performance OSCs.

Finally, the future of OSC design also involves addressing several critical questions that remain unanswered. These include understanding how nanoscale effects influence charge transport in OSCs and the relationship between transient localization and other excitonic processes like singlet fission. Advances in artificial intelligence and machine learning may also play a crucial role in identifying new candidate materials, not only enabling researchers to screen for high‐performance OSCs with tailored dynamic and structural properties, but also as a means of speeding up the calculation of lattice dynamics—not only at the gamma point, but across the entire Brillouin zone. These interdisciplinary efforts are essential for overcoming the current limitations and unlocking the full potential of OSCs in practical applications.

## 5 Summary and Outlook

This review highlights the intricate relationship between molecular dynamics and charge transport in OSCs. Understanding this interplay is proving critical to address the challenges posed by the modulation of electronic overlap due to nuclear motions, known as dynamic disorder. The role of low‐frequency vibrational modes in limiting charge carrier mobility, in particular, has enabled detailed insight into the fundamental origins of electron–phonon coupling in OSCs. Although advances in experimental techniques, such as terahertz and low‐frequency Raman spectroscopy, have significantly enhanced our understanding of these dynamics, computational methods like transient localization theory have enabled quantifying the effects of particular dynamics on charge carrier mobility in OSCs.

Recent studies have demonstrated that optimizing OSC performance requires precise manipulation of both molecular and crystal structures to mitigate the detrimental effects of these vibrational modes. However, such works have also highlighted the complexity introduced upon molecular modification, as the complex interplay between the equilibrium (static) configuration of atomic and molecular orbitals and the modulation of intermolecular overlap as a function of nuclear displacement implies that there are often numerous routes to modulate electronic coherence. Thus, although attempts at holistically stiffening specific molecular frameworks or controlling intermolecular interactions have certainly yielded great results, the next step in the rational design will involve directly optimizing intermolecular potential energy surfaces, the shape of which are what ultimately dictates the nature of resulting lattice dynamics. However, several challenges remain, including the computational cost of accurately simulating low‐frequency dynamics, and the gamma point and beyond, as well as the difficulty of predicting bulk crystal structures. Additionally, bridging the gap between molecular‐scale phenomena and practical device engineering continues to be a key objective for future research.

As this field progresses, a comprehensive approach that integrates experimental data with theoretical models will be essential for developing next‐generation organic electronics. Such efforts will not only push the boundaries of OSC design but also lay the groundwork for overcoming the limitations that currently constrain the performance of these materials. By advancing both our theoretical understanding and our ability to manipulate material properties at the atomic level, the future of OSCs remains promising for advanced applications in high‐performance electronics.

## Conflict of Interests

The authors declare no conflict of interest.

## Data Availability

Data sharing is not applicable to this article as no new data were created or analyzed in this study.
